# Quantifying the Oral Cancer Public Awareness Deficit in Germany (2015–2023)

**DOI:** 10.3390/cancers18081236

**Published:** 2026-04-14

**Authors:** Babak Saravi, Michael Vollmer, Daman Deep Singh, Lara Schorn, Julian Lommen, Felix Schrader, Max Wilkat, Andreas Vollmer, Veronika Shavlokhova, Marius Hörner, Norbert Kübler, Christoph Sproll

**Affiliations:** 1 Department of Oral, Maxillofacial and Facial Plastic Surgery, Medical Faculty and University Hospital Düsseldorf, Heinrich-Heine-University Düsseldorf, 40225 Düsseldorf, Germanychristoph.sproll@med.uni-duesseldorf.de (C.S.); 2 Department of Oral and Maxillofacial Surgery, Tübingen University Hospital, Osianderstrasse 2-8, 72076 Tuebingen, Germany; 3 Department of Oral and Maxillofacial Plastic Surgery, University Hospital of Würzburg, 97070 Würzburg, Germany; 4 Department of Oral and Maxillofacial Surgery, University Clinic Ruppin-Brandenburg, 16816 Neuruppin, Germany

**Keywords:** oral cancer, public awareness, Google Trends, Altmetric, COVID-19, infodemiology, Germany, health communication

## Abstract

**Objective:** To quantify the gap between oral cancer disease burden and public awareness in Germany, and to characterize research dissemination patterns across social media platforms. **Methods:** We conducted a multi-dimensional analysis integrating: (1) Robert Koch Institut cancer registry data for oral and maxillofacial malignancies (ICD-10: C00–C06) from 2015 to 2023; (2) Google Trends search interest for cancer-related German terms; (3) Altmetric data for 2581 PubMed-indexed oral cancer publications; and (4) sentiment analysis of 10,308 social media posts. Age-standardized incidence rates were calculated using the European Standard Population. **Results:** Over the study period, 65,757 oral cavity cancer cases were registered. Google Trends analysis revealed a 64% attention deficit for “Mundkrebs” (oral cancer; mean: 17) compared to “Brustkrebs” (breast cancer; mean: 47). Case numbers declined from 7577 (2019) to 6870 (2023; −9.3%), while age-standardized rates decreased by 15.5% (11.6 to 9.8 per 100,000), with males disproportionately affected (−17.7%). Research dissemination was dominated by X/Twitter (86.2%), with minimal policy document (0.3%) or clinical guideline (0.3%) citations. Sentiment analysis revealed 77% positive public reception. Regional analysis identified an East–West divide, with Eastern German states showing 22% higher search interest. **Conclusions:** A substantial public awareness deficit exists for oral cancer in Germany, paradoxically widening during a period of declining diagnoses potentially associated with COVID-19-related diagnostic delays. The positive public sentiment toward oral cancer research suggests a favorable environment for targeted awareness campaigns, particularly in Western German states where search interest is lowest. These findings have practical implications for designing regionally tailored awareness campaigns prioritizing anatomically specific terminology. Future research should evaluate the effectiveness of such targeted interventions and assess whether post-pandemic diagnoses present at more advanced stages.

## Simple Summary

Oral cancer is diagnosed in nearly 390,000 people worldwide annually, yet public awareness remains critically low compared to other malignancies. This study examined the gap between oral cancer disease burden and public attention in Germany from 2015 to 2023 using cancer registry data, Google Trends, and Altmetric indicators. We found that oral cancer receives 64% less public search interest than breast cancer despite substantially higher case fatality rates (5-year relative survival: ~55% vs. ~88%). Following the COVID-19 pandemic, new oral cancer diagnoses declined by 9.3%, with age-standardized rates falling 15.5%, suggesting possible missed or delayed diagnoses rather than true incidence reduction. Social media engagement with oral cancer research is overwhelmingly positive (77%), indicating public receptiveness to awareness campaigns. These findings highlight an urgent need for targeted oral cancer awareness initiatives in Germany.

## 1. Introduction

Oral cancer represents a significant global health burden, with an estimated 389,846 new cases and 188,438 deaths reported worldwide in 2022 [1]. Despite advances in diagnosis and treatment, five-year survival rates remain approximately 50%, largely attributable to late-stage presentation [2]. The primary modifiable risk factors—tobacco use, alcohol consumption, and human papillomavirus (HPV) infection—are well-established [3], yet public awareness of oral cancer and its warning signs remains critically low compared to other common malignancies [4,5].

Public awareness is a crucial determinant of cancer outcomes as knowledge of symptoms and risk factors facilitates earlier presentation and diagnosis [6,7]. Studies consistently demonstrate that delays in the diagnostic pathway for oral cancer are associated with poorer survival outcomes [8]. Unfortunately, surveys across multiple countries have revealed substantial knowledge gaps regarding oral cancer, with many individuals unable to identify common risk factors or early warning signs [9,10]. This awareness deficit is particularly concerning given that oral cancer can often be detected through routine visual examination of the oral cavity.

The COVID-19 pandemic introduced unprecedented disruptions to cancer care globally. A systematic review reported a 27% decrease in cancer diagnoses during 2020, with diagnostic delays potentially contributing to stage migration and increased mortality [11]. For oral cancer, which relies heavily on clinical examination and patient presentation, pandemic-related healthcare avoidance and reduced access to dental services may have had particularly pronounced effects [12]. Understanding the magnitude and persistence of these disruptions is essential for healthcare planning and resource allocation.

Google Trends has emerged as a validated infodemiology tool for assessing public interest in health topics [13,14,15]. By analyzing search query volumes, researchers can quantify temporal and geographic patterns of public attention to specific diseases, evaluate the effectiveness of awareness campaigns, and identify populations with unmet information needs [16,17]. Previous studies have demonstrated the utility of Google Trends for cancer awareness research, revealing significant disparities in public interest across different malignancies [16,18,19].

Complementing population-level awareness data, Altmetric indicators provide insights into research dissemination and engagement across social media and traditional media platforms [20]. The Altmetric Attention Score (AAS) quantifies online attention to scholarly outputs, tracking mentions across X/Twitter, news outlets, blogs, policy documents, and other sources [21]. While AAS shows only weak correlation with traditional citation metrics, it captures dimensions of research impact—particularly public engagement—that citations alone cannot measure [22,23]. Publications without Altmetric records were excluded from attention analyses but retained for citation-based analyses using iCite data. Publications lacking Altmetric data may represent articles with no social media or news mentions, introducing potential selection bias toward higher-visibility research.

Despite Germany’s well-developed healthcare system and comprehensive cancer registry, no study has systematically examined the gap between oral cancer disease burden and public awareness in this population. Furthermore, the research-to-practice translation pathway for oral cancer—how scientific findings reach policymakers and influence clinical guidelines—remains poorly characterized.

This study aimed to: (1) quantify the public awareness deficit for oral cancer compared to other malignancies in Germany; (2) characterize temporal trends in oral cancer incidence before and after the COVID-19 pandemic using age-standardized rates; (3) analyze patterns of oral cancer research dissemination across social media and traditional media platforms; and (4) identify regional disparities in oral cancer search interest across German federal states. By integrating cancer registry data, Google Trends, and Altmetric indicators, we provide a comprehensive assessment of the oral cancer attention gap and its implications for public health interventions.

## 2. Materials and Methods

### 2.1. Study Design

This multi-dimensional cross-sectional study integrated four complementary data sources to characterize the oral cancer attention gap in Germany: (1) national cancer registry incidence data; (2) Google Trends search interest data; (3) Altmetric attention indicators for oral cancer research publications; and (4) social media sentiment analysis. The primary study period spanned January 2015 to December 2023 for cancer registry and Google Trends data. To capture recent research dissemination patterns, Altmetric mention data were extracted through December 2024; this extended window is reported separately and does not affect the epidemiological or Google Trends analyses.

### 2.2. Cancer Registry Data

#### 2.2.1. Data Source

Oral cavity cancer incidence data were obtained from the Robert Koch Institut (RKI) Zentrum für Krebsregisterdaten (German Centre for Cancer Registry Data), which aggregates population-based cancer registration from all 16 German federal states [24]. Data were extracted for malignant neoplasms of the lip, oral cavity, and pharynx according to the International Classification of Diseases, 10th Revision (ICD-10). Only incident (first primary) tumors were included; recurrent cases were excluded as per standard cancer registry methodology. German cancer registry data are considered ≥90% complete from 2009 onward. A reporting lag of approximately two years may affect the most recent data points (2022–2023).

#### 2.2.2. Case Definition

The primary analysis focused on oral and maxillofacial surgery (OMFS)-relevant malignancies (hereafter referred to as ‘oral cavity cancer’ unless otherwise specified), defined as ICD-10 codes C00–C06 (Table 1):

Secondary analyses included pharyngeal sites (C09–C11, C14: tonsil, oropharynx, nasopharynx, and other/unspecified pharyngeal locations) to provide broader context.

**Table 1:** ICD-10 codes included in the primary analysis of oral and maxillofacial malignancies (OMFS).

ICD-10	Description
C00	Malignant neoplasm of lip
C01	Malignant neoplasm of base of tongue
C02	Malignant neoplasm of other and unspecified parts of tongue
C03	Malignant neoplasm of gingiva
C04	Malignant neoplasm of floor of mouth
C05	Malignant neoplasm of palate
C06	Malignant neoplasm of other and unspecified parts of mouth

#### 2.2.3. Age Standardization

Age-standardized incidence rates (ASRs) were obtained from the Robert Koch Institut and are based on the European Standard Population as the reference, expressed per 100,000 population [25]. Sex-specific rates represent the male and female contributions to the total age-standardized rate (i.e., calculated using the total population as denominator); consequently, they sum to the overall ASR. The male-to-female incidence ratio was calculated annually to assess temporal changes in sex disparity.

### 2.3. Google Trends Data

#### 2.3.1. Search Terms and Parameters

Google Trends (https://trends.google.com; accessed on 07 December 2025) data were extracted for Germany using German-language search terms relevant to oral cancer and comparator malignancies:

• “Mundkrebs” (oral cancer);
• “Zungenkrebs” (tongue cancer);
• “Brustkrebs” (breast cancer);
• “Hautkrebs” (skin cancer).

Additionally, ‘Corona’ was queried for the overlapping period to contextualize pandemic-related attention displacement. Data were retrieved for the period of 1 January 2015 to 31 December 2023 using the “Health” category filter to reduce noise from non-health-related queries [26]. Each term was queried independently to obtain term-specific relative search volume (RSV) values. To assess the robustness of our primary search terms, we systematically evaluated nine German-language terms related to oral cancer (Mundkrebs, Mundhöhlenkrebs, Mundschleimhautkrebs, Krebs im Mund, Zungenkrebs, Rachenkrebs, Lippenkrebs, Gaumenkrebs, Kieferkrebs). Pairwise comparison against “Mundkrebs” revealed that four terms (Mundschleimhautkrebs, Gaumenkrebs, Kieferkrebs, Lippenkrebs) had insufficient search volumes for reliable temporal analysis, while “Mundhöhlenkrebs” (ratio 0.72) was retained as an additional synonym for sensitivity analysis (Supplementary Table S1). Pairwise synonym comparisons were conducted without the Health category filter to ensure maximum term capture; the primary temporal and regional analyses retained the Health category filter as described above.

#### 2.3.2. Relative Search Volume

Google Trends provides relative search volume (RSV) data normalized to a 0–100 scale, where 100 represents peak search interest within the specified timeframe and geography. RSV values are normalized independently for each query; therefore, direct numerical comparisons across different search terms reflect relative rather than absolute search volumes and should be interpreted accordingly [26]. Monthly RSV values were extracted and annual means were calculated for trend analysis. For regional analysis, RSV data were extracted at the federal state (Bundesland) level for all 16 German states.

#### 2.3.3. Regional Classification

Federal states were classified as East Germany (Brandenburg, Mecklenburg-Vorpommern, Saxony, Saxony-Anhalt, Thuringia, and Berlin) or West Germany (all remaining states) to assess potential East–West disparities in oral cancer search interest.

### 2.4. Altmetric Data

#### 2.4.1. Publication Identification

PubMed (https://pubmed.ncbi.nlm.nih.gov; accessed on 7 December 2025) was searched using the query “oral cancer” OR “mouth cancer” OR “oral cavity cancer” OR “oral squamous cell carcinoma”, with publication dates restricted to 1 January 2015 through 31 December 2023. The search yielded 5996 publications.

#### 2.4.2. Altmetric Data Extraction

Altmetric data were retrieved via the Altmetric Explorer platform (https://www.altmetric.com/explorer/; accessed on 7 December 2025) using Digital Object Identifiers (DOIs) for all identified publications. The following metrics were extracted:

• Altmetric Attention Score (AAS);
• Platform-specific mention counts (X/Twitter, Facebook, news outlets, blogs, Wikipedia, policy documents, and clinical guidelines);
• First-author country affiliation;
• Open Access status.

Citation counts and Relative Citation Ratios (RCRs) were obtained from the NIH iCite database (https://icite.od.nih.gov; accessed on 7 December 2025) by matching PubMed identifiers (PMIDs). RCR is a field-normalized metric that benchmarks a publication’s citation rate against the median for NIH-funded papers in the same field and year. Of the 5996 publications, 2581 (43.0%) had Altmetric data available and were included in the dissemination analysis.

#### 2.4.3. Temporal Mention Analysis

Daily mention counts were extracted from Altmetric, enabling analysis of temporal patterns in research attention. Annual aggregations were computed for trend analysis.

### 2.5. Social Media Demographics and Sentiment Analysis

#### 2.5.1. X/Twitter Demographics

Geographic and demographic data for X/Twitter mentions were extracted from the Altmetric researcher group’s analysis module. Country of origin was determined based on user profile location data where available; posts without identifiable geographic information were classified as “Unknown.”

#### 2.5.2. Sentiment Analysis

Sentiment analysis—a natural language processing technique that classifies text into emotional categories (positive, negative, or neutral) based on linguistic features—was performed on 10,308 X/Twitter and Bluesky posts mentioning oral cancer research publications. Altmetric’s integrated sentiment classification algorithm categorized posts into five categories [27]:

• Strong positive;
• Positive;
• Neutral positive;
• Neutral;
• Negative (including neutral negative, negative, and strong negative).

For summary statistics, sentiments were aggregated into three categories: positive (strong positive, positive, and neutral positive combined), neutral, and negative (all negative categories combined). The classification was performed using Altmetric’s integrated sentiment analysis algorithm. This algorithm has not been specifically validated for medical or oncological discourse, and automated sentiment classification may misclassify domain-specific language (e.g., clinical terminology erroneously interpreted as negative sentiment).

### 2.6. Statistical Analysis

Descriptive statistics were calculated for all continuous variables (means, medians, standard deviations, and ranges) and categorical variables (frequencies and percentages). Temporal trends in cancer incidence were assessed by calculating absolute and percentage changes relative to the 2019 baseline (pre-COVID reference year).

Pearson correlation coefficients were calculated to assess relationships between OMFS cancer incidence and Google Trends RSV. Spearman correlation coefficients were calculated to assess relationships between Altmetric Attention Score and citation counts. Regional differences in Google Trends RSVs were compared between East and West German states using mean values and percentage differences. Platform distribution was summarized as absolute counts and percentages of total mentions. The “translation gap” was operationally defined as the proportion of mentions appearing in policy documents and clinical guidelines relative to total mentions.

All statistical analyses were performed using Python (version 3.10) and the pandas (version 2.0.3), numpy (version 1.24.3), and scipy (version 1.11.1) libraries. Visualizations were created using matplotlib (version 3.8.0) and seaborn (version 0.13.2). Statistical significance was set at *p* < 0.05 for correlation analyses. Given the exploratory nature of the correlation analyses involving a small number of pre-specified comparisons, no formal correction for multiple comparisons (e.g., Bonferroni) was applied; these results should be interpreted accordingly. Formal time-series modeling (e.g., ARIMA) was not applied given the limited number of annual data points (*n* = 9), which precludes reliable autoregressive parameter estimation. Instead, Mann–Kendall trend tests and linear regression were used to formally assess temporal trends in incidence data.

## 3. Results

### 3.1. Disease Burden Trends

#### 3.1.1. Absolute Case Numbers

Between 2015 and 2023, the Robert Koch Institut (RKI) registered a total of 65,757 new oral cavity cancer cases (ICD-10: C00–C06) in Germany. Mann–Kendall trend analysis confirmed a statistically significant declining trend in both absolute case numbers (S = −22, Z = −2.19, *p* = 0.029, Sen’s slope = −68.0 cases/year) and age-standardized incidence rates (S = −29, Z = −2.92, *p* = 0.004, Sen’s slope = −0.23/100,000/year). Annual case numbers showed a biphasic pattern: an initial increase from 7271 (2015) to a peak of 7577 (2019), followed by a sustained decline to 6870 cases in 2023 (Table 2). This represents a 9.3% decrease from the pre-COVID baseline year of 2019.

#### 3.1.2. Age-Standardized Incidence Rates

**Figure 1:**
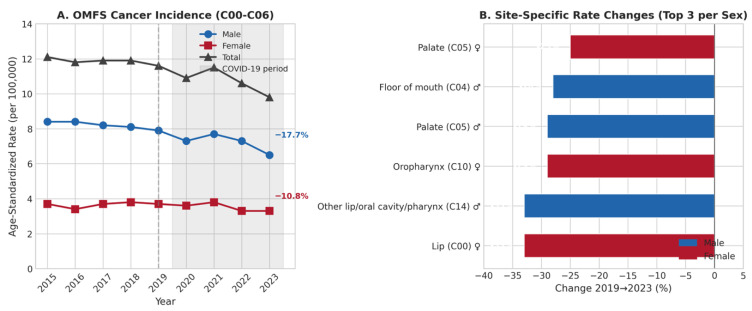
Age-standardized incidence rates of oral cavity cancer in Germany. (**A**) Temporal trends by sex, 2015–2023. (**B**) Site-specific rate changes between 2019 and 2023, displaying site-specific rate changes by sex.

Age-standardized incidence rates (European Standard Population) confirmed and amplified the observed decline (Table 3, Figure 1A). The total OMFS cancer ASR decreased from 11.6 per 100,000 (2019) to 9.8 per 100,000 (2023), representing a 15.5% decline—substantially larger than the 9.3% decline in absolute numbers. This difference indicates that the decline exceeds what demographic shifts alone could explain.

Sex-specific analysis revealed disproportionate male involvement: the male contribution to the total ASR declined by 17.7% (from 7.9 to 6.5 per 100,000) compared to 10.8% for the female contribution (from 3.7 to 3.3 per 100,000). The male-to-female incidence ratio progressively narrowed from 2.27:1 (2015) to 1.97:1 (2023), representing a 13.2% reduction in the sex disparity.

#### 3.1.3. Site-Specific Analysis

Site-specific analysis revealed heterogeneous decline patterns (Figure 1B). The largest declines between 2019 and 2023 were observed for:

• Floor of mouth (C04) in males: −28% (1.8 to 1.3 per 100,000);
• Palate (C05) in males: −29% (0.7 to 0.5 per 100,000);
• Lip (C00) in females: −33% (0.3 to 0.2 per 100,000);
• Oropharynx (C10) in females: −29% (0.7 to 0.5 per 100,000).

These site-specific patterns are consistent with the hypothesis of differential diagnostic delays, although alternative explanations cannot be excluded. Anatomical locations requiring detailed clinical examination showed the greatest decline.

### 3.2. Public Awareness Indicators

#### 3.2.1. The Attention Gap

**Figure 2:**
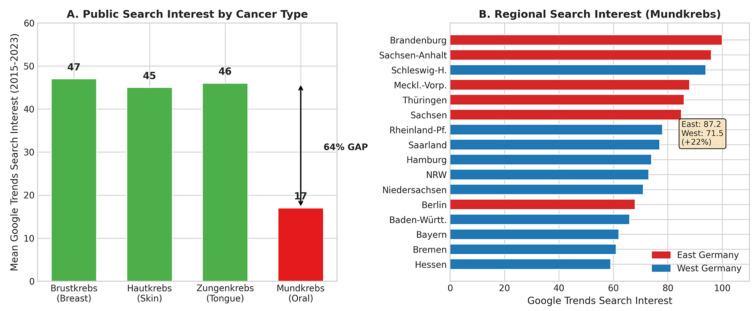
The oral cancer attention gap. (**A**) Mean Google Trends search interest by cancer type in Germany, 2015–2023. (**B**) Regional variation in “Mundkrebs” search interest by federal state.

Google Trends analysis revealed a substantial and persistent attention gap between oral cancer and other common malignancies in Germany (Figure 2A). Over the study period (2015–2023), the mean search interest for “Mundkrebs” (oral cancer) was 17, compared to 47 for “Brustkrebs” (breast cancer) and 45 for “Hautkrebs” (skin cancer). This represents a 64% attention deficit (2.7-fold difference) relative to breast cancer.

Notably, “Zungenkrebs” (tongue cancer) maintained a search interest comparable to breast cancer (mean: 46), suggesting that anatomically specific terminology may enhance public engagement. In 2023, tongue cancer search interest spiked to 53.9 (+18% above the historical average), potentially indicating successful awareness activities or media coverage for this specific subsite.

#### 3.2.2. Paradoxical Correlation with Disease Burden

Correlation analysis between OMFS cancer incidence and Google Trends search interest revealed a paradoxical inverse relationship:

• OMFS cases vs. “Mundkrebs”: r = −0.58 (95% CI: −0.91 to 0.06) (*p* = 0.099; exploratory) (moderate inverse correlation);
• OMFS cases vs. “Zungenkrebs”: r = −0.76 (95% CI: −0.94 to −0.14) (*p* = 0.013) (strong inverse correlation).

Notably, the correlation with ‘Mundkrebs’ did not reach statistical significance and should be considered exploratory. While the correlation with ‘Zungenkrebs’ reached statistical significance (*p* = 0.013), the ‘Mundkrebs’ correlation did not (*p* = 0.099), though both showed inverse trends. These negative correlations suggest that as disease burden increased (2015–2019), public search interest paradoxically did not follow, suggesting a widening rather than narrowing attention gap.

#### 3.2.3. Regional Disparities

Substantial regional variation in oral cancer search interest was observed across German federal states (Figure 2B, Table 4). Search interest ranged from 59 (Hessen) to 100 (Brandenburg), representing a 1.7-fold difference. A clear East–West divide emerged: Eastern German states (including Berlin) showed a mean search interest of 87.2 compared to 71.5 for Western states (+22% higher in the East).

#### 3.2.4. COVID-19 Attention Crowding

**Figure 3:**
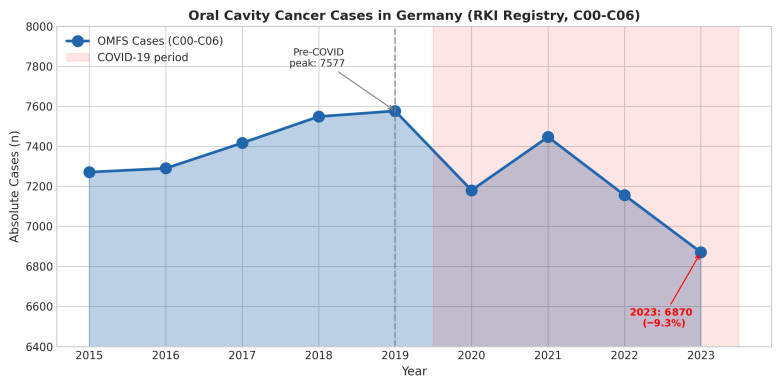
Impact of COVID-19 on oral cavity cancer diagnoses in Germany, 2015–2023. Annual absolute case numbers for oral and maxillofacial malignancies (ICD-10: C00–C06) from the Robert Koch Institut cancer registry. The dashed vertical line indicates the pre-COVID baseline year (2019; peak: 7577 cases). The shaded pink area represents the COVID-19 pandemic period (2020–2023), during which registered cases declined by 9.3% to 6870 cases in 2023, a pattern consistent with pandemic-related diagnostic disruption, although causality cannot be established from these data. This pattern was consistently observed across multiple cancer types in Germany: cancer registries nationwide reported significant declines in new diagnoses during the pandemic, driven by patients’ reluctance to seek care, prolonged diagnostic pathways, and temporary suspension of screening programs [28]. In Bavaria, incident malignancies declined by 6.7% overall, with reductions exceeding 10% for colorectal, skin, and liver cancers [29], while breast cancer diagnoses fell by approximately one-third during the first restriction period when mammography screening was suspended [28].

During the COVID-19 pandemic, oral cancer search interest was effectively “crowded out” by pandemic-related queries. In March 2020, “Corona” search interest peaked at 100 while “Mundkrebs” remained at baseline levels (1–2), rendering oral cancer awareness essentially invisible during a critical period when preventive healthcare visits declined substantially. This attention crowding coincided with a sustained decline in registered oral cancer cases, from the pre-COVID peak of 7577 (2019) to 6870 in 2023 (−9.3%), suggesting that reduced public awareness during the pandemic may have contributed to delayed diagnoses (Figure 3).

### 3.3. Research Dissemination and Social Media Engagement

#### 3.3.1. Publication Characteristics

**Figure 5:**
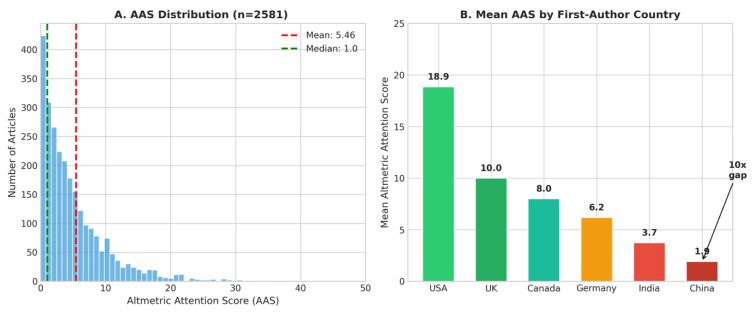
Altmetric Attention Score (AAS) analysis. (**A**) Distribution of AAS among 2581 oral cancer publications with Altmetric data, showing highly right-skewed distribution (mean: 5.46, median: 1.0). (**B**) Mean AAS by first-author country affiliation, demonstrating 10-fold difference between United States and China.

Of 5996 PubMed-indexed oral cancer publications retrieved, 2581 (43.0%) had Altmetric data available. Among articles with Altmetric coverage, the mean Altmetric Attention Score (AAS) was 5.46 (median: 1.0; range: 0–814), indicating a highly right-skewed distribution with most articles receiving minimal public attention (Figure 5A).

Correlation between AAS and traditional citation metrics was weak: Spearman’s ρ = 0.19 (*p* < 0.001) for AAS versus citation count, and ρ = 0.20 for AAS versus Relative Citation Ratio (RCR). This indicates that public attention and academic impact represent largely independent dimensions of research influence.

Open Access status significantly predicted higher attention: Open Access articles achieved mean AAS of 6.70 compared to 2.59 for non-Open Access articles, representing 2.6-fold higher attention.

#### 3.3.2. Platform Distribution

**Figure 4:**
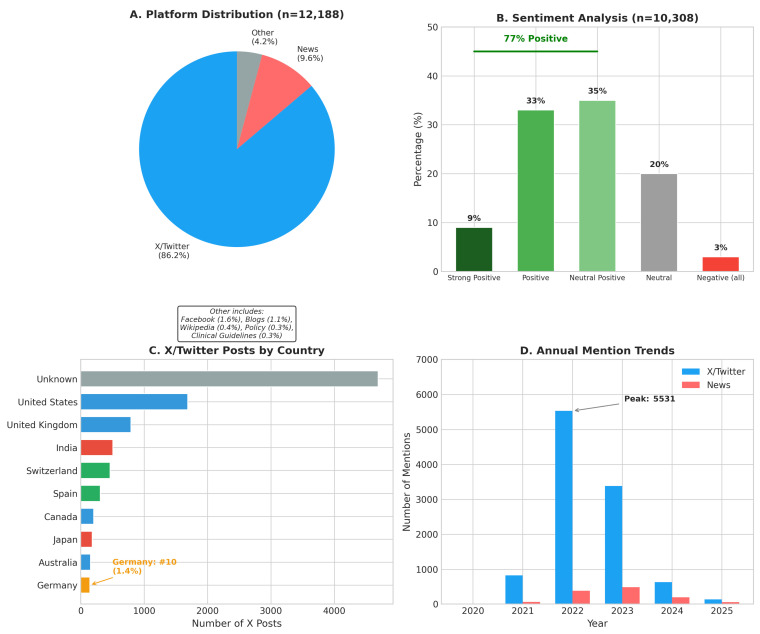
Altmetric analysis of oral cancer research dissemination. (**A**) Platform distribution of mentions. (**B**) Sentiment analysis of social media posts. (**C**) Geographic distribution of X/Twitter engagement. (**D**) Temporal trends in annual mentions.

Analysis of 12,188 total mentions across platforms revealed a pronounced hierarchy (Figure 4A, Table 5). X/Twitter dominated with 10,507 mentions (86.2%), followed by news media (1174; 9.6%), Facebook (197; 1.6%), and blogs (138; 1.1%).

Critically, policy document citations remained rare (33 mentions; 0.3%) and clinical guideline references were similarly scarce (38 mentions; 0.3%). This indicates a substantial translation gap: oral cancer research generates significant social media discussion but rarely reaches policymakers or influences clinical practice guidelines.

#### 3.3.3. Geographic Patterns of Engagement

X/Twitter demographic analysis identified 10,507 posts from 8216 unique profiles across 113 countries (Figure 4C). Geographic attribution was unavailable for 44.6% of posts. Among posts with a known origin, the United States (16.1%) and United Kingdom (7.5%) dominated, collectively accounting for 23.6% of all mentions.

Despite Germany being a major oral cancer research producer, German X/Twitter engagement ranked only 10th globally (143 posts; 1.4%), suggesting an English-language/Anglophone bias in research dissemination or lower social media engagement among German-speaking researchers and clinicians.

#### 3.3.4. Sentiment Analysis Results

Automated sentiment analysis of 10,308 X/Twitter and Bluesky posts revealed predominantly positive public reception (Figure 4B):

• Positive sentiment (including neutral-positive): 77%;
• Neutral: 20%;
• Negative (all categories): 3%.

This overwhelmingly positive sentiment suggests that the public is receptive to oral cancer research communications, providing a favorable foundation for awareness campaigns.

#### 3.3.5. Temporal Dynamics

Social media attention showed marked temporal variation (Figure 4D). X/Twitter mentions increased dramatically from 827 (2020–2021) to 8916 (2022–2023), representing a 978% increase, although part of this increase may reflect broader platform growth and algorithmic changes rather than solely genuine increased engagement with oral cancer research. Peak activity occurred in 2022 (5531 mentions), with notable spikes on 17–21 November 2022 (exceeding 140 daily mentions).

This paradox—increase in research attention while disease diagnoses decreased—suggests that the scientific community became increasingly active in discussing oral cancer research during a period when patients may have been missing by clinical detection.

#### 3.3.6. Geographic Research Output Disparities

The mean AAS varied substantially by first-author country (Figure 5B). United States-affiliated research achieved the highest attention (mean AAS: 18.86), followed by the United Kingdom (10.00), Canada (7.99), and Germany (6.17). Chinese-affiliated research, despite a high output volume (*n* = 640 articles), achieved the lowest mean attention score (1.91), representing a 10-fold difference compared to US research.

## 4. Discussion

This multi-dimensional analysis reveals a substantial and persistent public awareness deficit for oral cancer in Germany, characterized by a 64% attention gap relative to breast cancer, paradoxically widening during a period of declining diagnoses potentially associated with COVID-19-related diagnostic delays. Our findings integrate cancer registry epidemiology, infodemiological data, and Altmetric indicators to provide the first comprehensive assessment of the oral cancer attention gap in a major European healthcare system.

### 4.1. The Oral Cancer Attention Gap

The 2.7-fold difference in Google Trends search interest between oral cancer and breast cancer identified in this study is consistent with, yet more pronounced than, disparities reported in other national contexts. Cohen and colleagues demonstrated similar attention gaps across multiple cancer types in high-income countries, with breast cancer consistently dominating public search behavior [18]. Our findings extend this pattern to the German population, where the attention deficit persists despite substantially lower survival rates for oral cancer compared with breast cancer (5-year relative survival: approximately 55% vs. 88% according to German cancer registry data) [30,31].

The paradoxical inverse correlation between oral cancer incidence and public search interest (r = −0.58) warrants particular attention. Unlike breast cancer, where awareness campaigns have successfully linked disease burden to public engagement [32], oral cancer search behavior appears disconnected from epidemiological trends. This finding aligns with Kaminski et al.’s systematic review, which noted that Google Trends data for certain cancers do not necessarily correspond to clinical occurrence but may instead reflect media coverage, celebrity diagnoses, or awareness campaign timing [15].

Notably, the subsite-specific term “Zungenkrebs” (tongue cancer) maintained a search interest comparable to breast cancer, suggesting that anatomically specific terminology may enhance public engagement. A systematic evaluation of nine German-language search terms confirmed this pattern, with ‘Zungenkrebs’ achieving a 2.65-fold higher RSV than ‘Mundkrebs’ (Table S1). This observation has practical implications for awareness campaign design: terminology selection may be as important as campaign reach in determining public response.

The disparity in public attention may also partly reflect structural differences in pharmaceutical industry engagement and advocacy funding across cancer types. Breast cancer is the most heavily funded malignancy globally: in the United States, the National Cancer Institute allocates over $540 million annually to breast cancer research, resulting in a funding-to-lethality ratio more than tenfold higher than most other cancers [33]. Nonprofit organizations direct a third of all cancer-specific revenue to breast cancer alone [34]. These investments generate sustained public awareness campaigns that further reinforce the attention advantage. In contrast, cancers linked to stigmatized risk behaviors—such as tobacco and alcohol use in oral and oropharyngeal cancer—have been shown to be systematically underfunded, as fewer long-term survivors are available to serve as advocates [34]. However, this funding landscape may be shifting: the approval and expanding indications of immune checkpoint inhibitors (ICIs) for head and neck squamous cell carcinoma—particularly pembrolizumab as a first-line treatment for recurrent/metastatic disease [35] and, more recently, in curative-intent perioperative settings [36]—are driving substantial pharmaceutical investment in HNSCC.

### 4.2. COVID-19 and Diagnostic Disruption

The 9.3% decline in oral cavity cancer cases and 15.5% reduction in age-standardized incidence rates from 2019 to 2023 may partly reflect diagnostic delays rather than a true incidence reduction, although true incidence changes cannot be fully excluded. This interpretation is supported by three observations: first, the decline magnitude substantially exceeds demographic shifts; second, anatomical sites requiring detailed clinical examination showed the greatest reductions; and third, the pattern is consistent with international reports of COVID-19-related cancer diagnostic delays [37,38].

Our findings corroborate multicenter German data that reported that closure of dental practices during lockdown periods delayed oral cancer diagnosis [39]. Similarly, Arduino et al. documented worrying diagnostic delays in Northern Italy during the pandemic’s first wave [40]. Treatment delay studies have demonstrated significantly longer intervals from presentation to surgery during 2020 compared to pre-pandemic periods, with associated increases in tumor size at presentation [41].

The disproportionate decline in male cases (−17.7% vs. −10.8% in females) may reflect differential healthcare-seeking behavior, as men have been shown to delay medical consultations more frequently than women [42]. The narrowing male-to-female incidence ratio (from 2.27:1 to 1.97:1) during this period likely represents differential diagnostic delays rather than genuine epidemiological convergence.

The site-specific analysis revealed that floor of mouth carcinomas showed the largest decline (−28% in males), consistent with the hypothesis that lesions requiring detailed clinical examination—typically performed during routine dental visits—experienced the greatest diagnostic disruption. This has significant prognostic implications: modeling studies suggest that even modest diagnostic delays can substantially reduce survival, with estimates of 12–18% reductions in 10-year survival for each 6-month delay [43].

### 4.3. Regional Disparities: The East–West Divide

The 22% higher oral cancer search interest in Eastern German states represents an unexpected finding that inverts traditional East–West health gradients. Historically, Eastern Germany has exhibited higher cancer mortality, particularly for smoking-related malignancies, attributable to higher tobacco consumption, socioeconomic disadvantage, and the legacy of different healthcare systems prior to reunification [44,45].

Several hypotheses may explain this paradox. First, the higher disease burden in Eastern Germany may drive greater information-seeking behavior among affected populations and their networks. Recent data confirm persistent survival disadvantages for head and neck cancer patients in Eastern Germany, with residence in the East confirmed as an independent prognostic factor in multivariate analyses [46]. Second, regional variation in healthcare utilization patterns may influence search behavior; notably, screening uptake for cervical cancer and mammography is actually higher in Eastern Germany, potentially reflecting cultural differences in health engagement stemming from the former GDR’s preventive medicine emphasis [47].

Third, socioeconomic factors may paradoxically increase health-information seeking in disadvantaged populations facing limited healthcare access. The 30-year trajectory toward equalization of health outcomes between East and West Germany has stalled in recent years for some indicators [48], suggesting that awareness campaigns may need to prioritize different regions than what traditional health intervention models would suggest.

### 4.4. Research Dissemination and the Translation Gap

The Altmetric analysis revealed a substantial translation gap between oral cancer research dissemination and policy influence. While X/Twitter dominated attention (86.2%), policy document citations (0.3%) and clinical guideline references (0.3%) remained negligible. This pattern is consistent with broader findings that social media attention and traditional academic impact represent largely independent dimensions of research influence [49,50].

The weak correlation between Altmetric Attention Score and citation metrics (rho = 0.19) observed in our analysis aligns with meta-analytic evidence reporting pooled correlations of similar magnitude across the health sciences literature [51,52]. This suggests that oral cancer research achieving high public visibility does not necessarily translate to academic influence or clinical implementation.

The 2.6-fold higher attention for Open Access publications has practical implications for maximizing research impact. Given that awareness campaigns may benefit from accessible scientific communication, prioritizing Open Access dissemination could enhance both public engagement and policy influence.

The predominantly positive sentiment (77%) toward oral cancer research on social media suggests public receptiveness to scientific communication. This finding provides an evidence base for awareness campaigns: the audience is engaged and responsive, but the message is not reaching them with sufficient frequency or visibility.

### 4.5. Implications for Public Health Practice

Our findings have several practical implications. First, the attention gap suggests that existing awareness efforts are insufficient [53]. Germany does participate in the European Head and Neck Society’s (EHNS) Make Sense Campaign [7], which holds an annual Head and Neck Cancer Awareness Week each September (https://makesensecampaign.eu (accessed on 7 December 2025)), coordinated nationally by the Interdisziplinäre Arbeitsgruppe Kopf-Hals-Tumoren (IAG-KHT) of the Deutsche Krebsgesellschaft with over 20 participating clinics. However, this initiative has not yet achieved a sustained public visibility comparable to breast cancer awareness campaigns or dedicated national awareness months in the UK and US [53], and participation across German clinical centers remains inconsistent. The success of breast cancer awareness campaigns in generating sustained public interest demonstrates that such efforts can be effective when adequately resourced and strategically designed [32].

Second, our regional analysis suggests that awareness campaigns should prioritize Western German states, where search interest is 22% lower despite comparable or higher disease burdens. Targeted interventions in low-awareness regions may yield greater marginal benefits than uniform national campaigns.

Third, the COVID-19-related diagnostic disruption highlights the need for catch-up screening initiatives. If a proportion of the observed decline reflects delayed diagnoses, these cases may present at more advanced stages, requiring healthcare system preparation for increased treatment complexity and resource requirements.

Fourth, the effectiveness of subsite-specific terminology (“Zungenkrebs” vs. “Mundkrebs”) suggests that awareness campaigns might benefit from anatomically precise messaging rather than generic “oral cancer” terminology.

### 4.6. Strengths and Limitations

However, several limitations warrant consideration. First, Google Trends provides relative rather than absolute search volumes, precluding direct quantification of search frequency [14]. Moreover, Google Trends measures search behavior rather than awareness [15]; elevated search interest may reflect curiosity, anxiety, or information-seeking behavior rather than genuine understanding of the disease. Second, as an ecological study, aggregate-level associations between search interest and disease burden cannot be extrapolated to individual-level awareness or behavior, and the ecological nature of regional analyses precludes individual-level inferences about search behavior determinants. Third, Altmetric coverage is incomplete (43% of publications), potentially overrepresenting articles with higher social media visibility and underrepresenting research disseminated through non-digital channels, thereby biasing estimates of research dissemination patterns. Fourth, the sentiment analysis algorithm has not been specifically validated for health research contexts, and automated classification may miss nuanced discourses [54]. Fifth, the substantial increase in X/Twitter mentions during 2022–2023 may partially reflect platform-wide shifts in academic social media engagement following the COVID-19 pandemic and changes to Twitter’s API infrastructure, rather than oral cancer-specific trends alone. Sixth, a linguistic bias exists between the German-language Google Trends analysis and the predominantly English-language Altmetric and publication data, limiting direct comparability of awareness and dissemination metrics. Finally, the attribution of declining cancer diagnoses to COVID-19 disruption, while consistent with international evidence, cannot be definitively confirmed without individual-level diagnostic pathway data. Alternative explanations, including genuine incidence changes due to risk factor modification, cannot be entirely excluded.

### 4.7. Future Directions

Future research should evaluate the effectiveness of targeted awareness interventions in low-attention regions, assess whether post-pandemic diagnoses present at more advanced stages as predicted, and develop validated instruments for measuring oral cancer awareness at the population level. Longitudinal follow-up of the 2020–2023 cohort will be essential for quantifying the survival impact of diagnostic delays.

Additionally, comparative analyses with other European countries could identify successful awareness strategies that are potentially transferable to the German context. The positive social media sentiment identified in this study suggests favorable conditions for digital awareness campaigns, warranting evaluation of social media-based intervention effectiveness.

## 5. Conclusions

This study provides the first comprehensive quantification of the oral cancer public awareness deficit in Germany, revealing a 64% attention gap compared to breast cancer that persists despite substantially higher case fatality for oral cancer (5-year relative survival: ~55% vs. ~88%). The integration of cancer registry data, Google Trends analysis, and Altmetric indicators demonstrates that this awareness deficit paradoxically widened during 2020–2023, a period characterized by a 9.3% decline in new diagnoses and a 15.5% reduction in age-standardized incidence rates—patterns suggestive of COVID-19-related diagnostic delays rather than true incidence reduction. The disproportionate decline in cancers of anatomical sites requiring detailed clinical examination, particularly floor of mouth carcinomas (-28%), underscores the vulnerability of oral cancer detection to healthcare system disruptions.

Our findings identify actionable targets for public health intervention: Western German states exhibit 22% lower search interest than Eastern states and should be prioritized for awareness campaigns; subsite-specific terminology (“Zungenkrebs”) generates substantially higher engagement than generic terms (“Mundkrebs”); and the predominantly positive public sentiment toward oral cancer research (77%) indicates a receptive audience for educational initiatives. The substantial translation gap—with only 0.3% of research mentions appearing in policy documents or clinical guidelines despite high social media engagement—highlights the need for deliberate strategies to bridge scientific discovery and clinical implementation. Strengthening and expanding Germany’s participation in the existing European Make Sense Campaign—through sustained year-round funding, broader clinical participation beyond the current annual awareness week, and integration into primary care and dental practice networks—represents a concrete, evidence-informed policy recommendation to close the identified awareness gap. These findings should be interpreted within the constraints of ecological study design and validated through prospective studies incorporating individual-level data.

## Figures and Tables

**Table 2:** Annual oral cavity cancer cases (C00–C06) in Germany, 2015–2023. Source: Robert Koch Institut, Zentrum für Krebsregisterdaten. Change calculated relative to 2019 baseline.

Year	Male	Female	Total	Change
2015	5161	2110	7271	—
2016	5196	2094	7290	—
2017	5241	2176	7417	—
2018	5306	2243	7549	—
2019	5347	2230	7577	Baseline
2020	5040	2140	7180	−5.2%
2021	5252	2195	7447	−1.7%
2022	5045	2111	7156	−5.6%
2023	4791	2079	6870	−9.3%

**Table 3:** Age-standardized incidence rates of oral cavity cancer (C00–C06) per 100,000 population in Germany, 2015–2023. Rates were standardized to the European Standard Population and are presented as sex-specific contributions to the total age-standardized rate; consequently, male and female values sum to the total. Source: Robert Koch Institut, Zentrum für Krebsregisterdaten.

Year	Male	Female	Total	M:F Ratio
2015	8.4	3.7	12.1	2.27:1
2016	8.4	3.4	11.8	2.47:1
2017	8.2	3.7	11.9	2.22:1
2018	8.1	3.8	11.9	2.13:1
2019	7.9	3.7	11.6	2.14:1
2020	7.3	3.6	10.9	2.03:1
2021	7.7	3.8	11.5	2.03:1
2022	7.3	3.3	10.6	2.21:1
2023	6.5	3.3	9.8	1.97:1

**Table 4:** Google Trends search interest for “Mundkrebs” by German federal state, 2015–2023. Values represent relative search interest (0–100 scale).

Federal State	Search Interest	Region
Brandenburg	100	East
Sachsen-Anhalt	96	East
Schleswig-Holstein	94	West
Mecklenburg-Vorpommern	88	East
Thüringen	86	East
Sachsen	85	East
Rheinland-Pfalz	78	West
Saarland	77	West
Hamburg	74	West
Nordrhein-Westfalen	73	West
Niedersachsen	71	West
Berlin	68	East *
Baden-Württemberg	66	West
Bayern	62	West
Bremen	61	West
Hessen	59	West

* Berlin was classified as East Germany based on its historical status as the former German Democratic Republic (GDR) capital; however, this classification may not fully capture the city’s unique post-reunification demographic composition.

**Table 5:** Distribution of oral cancer research mentions by platform. Highlighted rows indicate channels for policy and clinical translation.

Platform	Mentions (n)	Percentage
X/Twitter	10,507	86.2%
News media	1174	9.6%
Facebook	197	1.6%
Blogs	138	1.1%
Wikipedia	47	0.4%
Clinical guidelines	38	0.3%
Policy documents	33	0.3%
Other	54	0.4%
Total	12,188	100%

## Data Availability

RKI cancer registry data are publicly available at https://www.krebsdaten.de (accessed on 7 December 2025). Google Trends data are publicly accessible at https://trends.google.com (accessed on 7 December 2025). Altmetric data were retrieved via an institutional subscription to Altmetric Explorer; aggregated data are available from the corresponding author on request. PubMed publication metadata are publicly available at https://pubmed.ncbi.nlm.nih.gov (accessed on 7 December 2025).

## Supplementary Materials

The following supporting information can be downloaded at: https://www.mdpi.com/article/10.3390/cancers18081236/s1, Table S1. Systematic evaluation of German-language search terms related to oral cancer using Google Trends (2015–2023). Relative search volume (RSV) ratios were calculated by pairwise comparison of each term against "Mundkrebs" (reference term, ratio = 1.00). Active months indicate the number of months (out of 108 total) in which the search term registered a non-zero RSV. Terms were retained for analysis if they demonstrated a sufficient and consistent search volume for reliable temporal trend assessment.
