# Supplementary material for: Quantifying the Oral Cancer Public Awareness Deficit in Germany (2015–2023)
Source: Cancers (Basel). 2026 Apr 14;18(8):1236. doi: 10.3390/cancers18081236 (PMC13114737; doi:10.3390/cancers18081236)
Supplement: Supplementary file 1 [file cancers-18-01236-s001.zip › cancers-4207736-supplementary.pdf]

Supplementary Materials

Table S1. Systematic evaluation of German-language search terms related to oral cancer using Google Trends (2015–2023). Relative search volume (RSV) ratios were calculated by pairwise comparison of each term against "Mundkrebs" (reference term, ratio = 1.00). Active months indicate the number of months (out of 108 total) in which the search term registered a non-zero RSV. Terms were retained for analysis if they demonstrated a sufficient and consistent search volume for reliable temporal trend assessment.

Search Term	English Translation	Category	Ratio to "Mundkrebs"	Active Months (of 108)	Retained for Analysis	Justification
Mundkrebs	Oral cancer	Primary term	1.00 (reference)	108	**Yes**	Primary search term; highest volume among oral cancer-specific terms
Mundhöhlenkrebs	Oral cavity cancer	Medical synonym	0.72	108	**Yes (sensitivity)**	Second-highest volume; retained for sensitivity analysis
Zungenkrebs	Tongue cancer	Anatomical subsite	2.65	108	**Yes**	Highest RSV of all terms; key finding on anatomical terminology
Krebs im Mund	Cancer in the mouth	Colloquial	0.33	106	No	Insufficient volume for reliable trend analysis
Rachenkrebs	Throat/pharyngeal cancer	Anatomical (pharynx)	1.02	108	No	Pharyngeal site; outside primary analysis scope (C00–C06)
Lippenkrebs	Lip cancer	Anatomical subsite	0.37	107	No	Insufficient volume for reliable trend analysis
Kieferkrebs	Jaw cancer	Anatomical subsite	0.26	105	No	Insufficient volume for reliable trend

						analysis
Gaumenkrebs	Palate cancer	Anatomical subsite	0.10	61	No	Very low and inconsistent volume (57% months active)
Mundschleimhautkrebs	Oral mucosa cancer	Medical synonym	0.01	3	No	Negligible search volume (3% months active)
Brustkrebs	Breast cancer	Comparator	37.35	108	**Yes**	Comparator malignancy for attention gap quantification
Hautkrebs	Skin cancer	Comparator	42.45	108	**Yes**	Comparator malignancy for attention gap quantification

Notes: RSV ratios were obtained via pairwise Google Trends queries pairing each term with "Mundkrebs" for Germany (2015–2023) without category filter. Due to Google Trends' independent normalization of each query, RSV values from separate queries are not directly comparable; pairwise comparison provides a reliable relative measure. Regional analysis confirmed that the East–West disparity in search interest observed for "Mundkrebs" was replicated across all tested synonyms (Mundhöhlenkrebs, Zungenkrebs, Rachenkrebs), with Eastern German federal states consistently ranking highest.
